# The Use of Psychotropic Medications Before and During the COVID-19 Pandemic and Its Associated Factors

**DOI:** 10.3390/jcm13237419

**Published:** 2024-12-05

**Authors:** Mohammed M. Alsultan

**Affiliations:** Department of Pharmacy Practice, College of Clinical Pharmacy, Imam Abdulrahman Bin Faisal University, Dammam 34212, Saudi Arabia; mmaalsultan@iau.edu.sa

**Keywords:** prescription, psychotropic, antidepressants, antipsychotics, anxiolytic-sedative/hypotonic, COVID-19

## Abstract

**Background/Objectives**: The prevalence of mental health disorders has been rising in Saudi Arabia, which may have been exacerbated by the COVID-19 pandemic. Therefore, the aim of our study was to examine the usage patterns of various psychotropic drugs before and during the pandemic. **Methods**: This cross-sectional study was conducted at the psychiatric outpatient clinic of a single hospital in Saudi Arabia from 1 October 2018 to 31 March 2023. Electronic medical records were used to gather information on all adult patients who were prescribed at least one antidepressant, antipsychotic, or anxiolytic/sedative/hypnotic medication. The data were analyzed using descriptive statistics and multivariable logistic regression model. **Results**: In the 4846 participants in the study, the total frequently prescribed psychotropics during the pandemic were antidepressants (2119 prescriptions), then antipsychotics (1509 prescriptions), and anxiolytics/sedatives/hypnotics (780 prescriptions). The mean before and during the pandemic for olanzapine was (41.86 vs. 23.55) and risperidone was (39.00 vs. 22.18), indicating a significant difference for both medications (*p* = 0.0003). Psychotropic drug use during the COVID-19 pandemic was significantly higher among the female patients (OR = 1.15, 95% CI [1.06–1.26]) and those aged 18–39 years (OR = 1.65, 95% CI [1.52–1.80]). Antidepressant and antipsychotic use were significantly lower than anxiolytic/sedative/hypnotic use during the pandemic (OR = 0.74, 95% CI [0.65–0.84]; OR = 0.66, 95% CI [0.58–0.75], respectively). **Conclusions**: The prescription rate of anxiolytics/sedatives/hypnotics was higher than that of antidepressants and antipsychotics. Furthermore, women and individuals aged ≤40 years were at a higher risk of psychotropic medication use. To mitigate stress, anxiety, and depression in Saudi Arabia, policymakers should implement mental health screening initiatives.

## 1. Introduction

The World Health Organization (WHO) designated the coronavirus disease (COVID-19) outbreak a worldwide pandemic after it quickly spread around the world from its origin in China [1,2]. This led to most countries taking precautions to curb the spread of the virus [3]. In Saudi Arabia (SA), the first COVID-19 case was reported in March 2020, prompting the country to implement several measures to stem the spread, including isolation and reducing social interactions [4,5,6,7,8]. The Saudi government was among the first to impose travel restrictions in an attempt to curb the spread of the virus because there was no traditional therapy or vaccine available at the beginning of the pandemic [4].

Although government restrictions will assist in preventing the spread of the virus, they may have a negative impact on the global economy [9]. This effect will lead to market instability and decreased industry activity as people will lose their jobs. Therefore, this may adversely affect a country’s ability to meet its citizens’ daily needs, especially those that rely heavily on overseas commerce [10,11]. Also, these negative effects were disseminated throughout families and society due to the sudden lockdown [12]. In emergency situations, the worldwide gross domestic product (GDP) is predicted to fall by 2.5 to 3% every month, on average. The median GDP during the spread of the COVID-19 infection dropped by around 2.8% [9,13]. Therefore, the healthcare system globally was significantly affected during the pandemic, with a decline in the use of healthcare services for urgent medical needs [14]. The cost of treatment and laboratory testing impacted the healthcare system in SA, affecting direct medical expenditures during the COVID-19 pandemic [15]. Consequently, the worldwide economic disaster may have caused mental health issues that have detrimental effects on individuals’ health [16,17].

In the United States (US) and Germany, the prevalence of mental health conditions such as depression and anxiety increased during the pandemic compared to that pre-pandemic [18,19]. Similarly, the initial spread of COVID-19 had a notable negative impact on mental health in SA, and mental health disorders became more common among the Saudi population due to the pandemic [20,21,22]. Furthermore, emergency room visits for mental health illnesses in China and the US surged substantially when compared to pre-pandemic levels [23,24]. In Canada, the use of various prescribed medications during the pandemic was documented to be lower than before the pandemic [25]. However, a study showed that the quantity of psychotropic medications increased during the pandemic among Danish teenagers [26]. Further, the use of antidepressants and antipsychotics in the same country increased for the first time among women and those aged 40 years and older during the first year of the pandemic [27]. In European nations and the Middle East, antidepressant use was higher during the pandemic period [28,29,30,31]. The mean day’s supply of psychotropic drugs in the US increased remarkably through the pandemic [32]. Furthermore, hospitalized patients were prescribed psychotropic drugs at a greater rate than those who visited ambulatory care settings prior to the pandemic in SA [33].

According to previous research, the global increase in psychotropic medication use during the COVID-19 pandemic may have led to health issues that still need to be addressed by healthcare professionals. In SA, the use of psychotropic drugs before and during the COVID-19 pandemic has not yet been studied. This study is crucial because the pandemic increased the prevalence of depression and stress among people in SA, highlighting the detrimental effects of this event on mental health. Moreover, the findings will have a significant impact on improving patient outcomes. Therefore, this study aimed to compare the use of psychotropic drugs before and during the COVID-19 pandemic and ascertain the associated predictors in SA.

## 2. Materials and Methods

### 2.1. Study Design and Setting

This observational, retrospective, cross-sectional study was conducted at the outpatient psychiatric clinic of King Fahad University Hospital in Al Khobar, SA.

### 2.2. Study Population

This study included individuals who were at least 18 years old and received at least one psychotropic medicine prescription (from October 2018 to the end of March 2023). These drugs were categorized as follows: (1) antidepressants: selective serotonin reuptake inhibitors (SSRIs) such as escitalopram and sertraline, serotonin and norepinephrine reuptake inhibitors (SNRIs) such as venlafaxine, tricyclics such as amitriptyline and clomipramine, and tetracyclics such as mirtazapine; (2) antipsychotics: atypical agents such as clozapine, olanzapine, paliperidone, quetiapine, and risperidone, and typical drugs such as haloperidol; and (3) anxiolytics/sedatives/hypnotics: benzodiazepines such as clonazepam, diazepam, and lorazepam, and non-benzodiazepines such as zolpidem. Patients who received psychotropic drugs in inpatient settings and those with incomplete information were excluded.

### 2.3. Data Source and Extraction

Data were extracted from the electronic medical record (EMR) database, covering the period from 1 October 2018 to 31 March 2023. We further classified the year into four quartets based on the months as: Q1 = quarter 1 (from January to March), Q2 = quarter 2 (from April to June), Q3 = quarter 3 (from July to September), and Q4 = quarter 4 (from October to December). The dataset included information on patient age, sex, and medication usage.

### 2.4. Study Variables

The primary outcome variable was COVID-19 status, categorized as before (Q4 2018 to Q1 2020) or during (Q2 2020 to Q1 2023) the pandemic, based on the government’s declaration of the pandemic in SA [4,5]. Covariates included demographic data (age: 18–39 vs. ≥40, sex: female vs. male), while the class of psychotropic medications (antidepressants, antipsychotics, and anxiolytics/sedatives/hypnotics) was our independent variable.

### 2.5. Data Analysis

Descriptive statistical analysis was performed to determine the baseline characteristics of patients using psychotropic medications, demonstrating frequencies and percentages. Trends were represented by the total number of classes per quarter of the year. The Shapiro–Wilk test was used to check data normality. Student’s *t*-test or the Wilcoxon signed-rank test was applied to determine significant mean differences in drug use before and during the COVID-19 pandemic. Multivariable logistic regression analysis was conducted to identify associated factors during the COVID-19 pandemic (dependent variable) after adjusting for age, sex, and classes of psychotropic medications along with the Hosmer–Lemeshow test to assess model fit. The odds ratio (OR) and confidence interval at 95% (95% CI) were reported. A *p*-value < 0.05 was considered statistically significant. All statistical analyses were performed using SAS software (version 9.4; SAS Institute Inc., Cary, NC, USA), and graphs were created using Microsoft^®^ Excel version 16.9.

## 3. Results

This study included data from 4846 patients from 1 October 2018 to 31 March 2023. The majority of the participants were women 55.08%, whereas men accounted for 44.92% of the sample. Patients aged 18–39 years comprised 52.81% of the sample, with those aged 40 and older making up 47.19%. Antidepressants were the most commonly used psychotropic drugs 47.73%, followed by antipsychotics 36.45%, and anxiolytics/sedatives/hypnotics were the least used 15.82%. More details are illustrated in Table 1.

**Figure 1:**
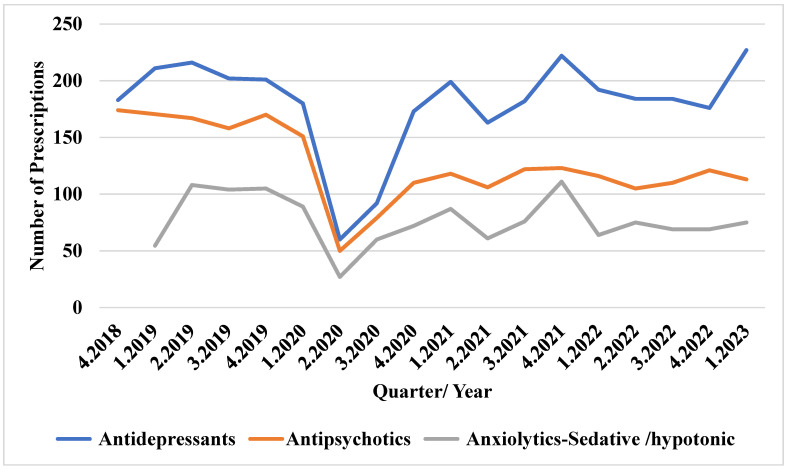
Trends in antidepressant, antipsychotic, and anxiolytic/sedative/hypnotic use before and during the COVID-19 pandemic.

Figure 1 shows the antidepressant use before the COVID-19 pandemic, with over 170 prescriptions per quarter from Q4 2018 to Q1 2020. However, usage dropped dramatically to approximately 50 prescriptions in Q2 2020, before gradually increasing and peaking at around 227 prescriptions in Q1 2023. The number of antipsychotic prescriptions exceeded 150 before the pandemic but dropped rapidly in Q2 2020, with the subsequent usage not surpassing 130 prescriptions in the 2 years following it. The anxiolytic/sedative/hypnotic prescriptions hovered around 100 per quarter from Q2 2019 to Q4 2019 but declined to fewer than 30 in Q2 2020, peaking again in Q4 2021 during the pandemic.

**Figure 2:**
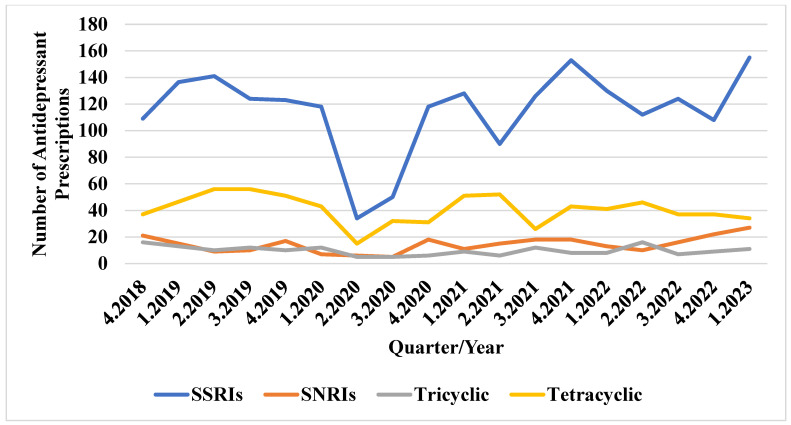
Trends in antidepressant use before and during the COVID-19 pandemic.

**Figure 3:**
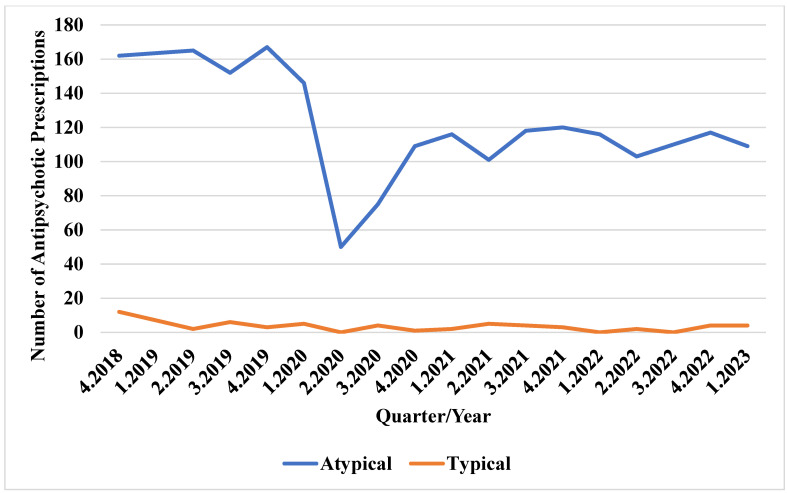
Trends in antipsychotic use before and during the COVID-19 pandemic.

As depicted in Figure 2, there were approximately 100 SSRI prescriptions per quarter from Q4 2018 to Q1 2020. The number of prescriptions dropped to below 40 in Q2 2020 but gradually increased during the pandemic, reaching 155 prescriptions in Q1 2023. The SNRI usage ranged between 40 and 60 prescriptions per quarter before and throughout the pandemic, dropping to around 20 in Q2 2020. The use of tricyclic and tetracyclic antidepressants remained consistent before and during the pandemic. Figure 3 indicates that atypical antipsychotic prescriptions peaked at 167 in Q4 2019 but sharply declined to about 50 in Q2 2020, gradually increasing again during the pandemic. The typical antipsychotic usage remained consistent at fewer than 12 prescriptions per quarter before and during the pandemic.

**Figure 4:**
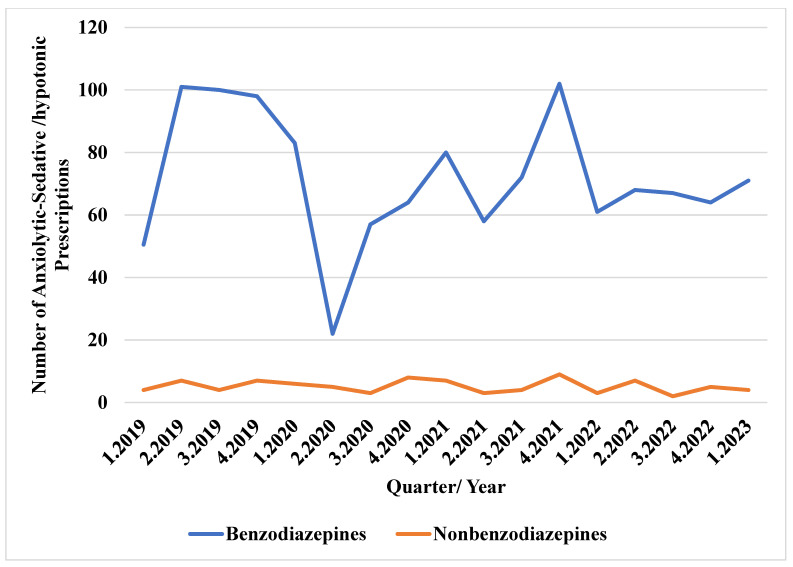
Trends of anxiolytic/sedative/hypnotic medication use before and during the COVID-19 pandemic.

Figure 4 shows that there were around 100 benzodiazepine prescriptions in Q2 2019, but this dropped sharply to approximately 25 in Q2 2020. This trend increased during the pandemic, peaking in Q4 2021, after which point its usage ranged around 65 prescriptions. The use of non-benzodiazepines remained relatively unchanged before and after the pandemic, with a maximum of 10 prescriptions per quarter.

The average number of prescriptions of SSRI antidepressants, such as escitalopram, prior to the pandemic was around 77.57, but this has subsequently decreased by more than 75% to around 20.50. However, the average number of sertraline prescriptions was observed to be 47.00, which is higher than it was before the pandemic, at 34.64. The change in escitalopram and sertraline use was not statistically significant between the period before and during the pandemic. The mean use of venlafaxine, which was classified as an SNRI, did not vary significantly between the periods before and during the pandemic. The average use of amitriptyline, clomipramine, and mirtazapine was not significantly different before and during the pandemic (*p*-value > 0.05).

The mean number of prescriptions of the atypical antipsychotic olanzapine prior to the pandemic was 41.86, but it decreased dramatically to roughly 23.55 during the pandemic period (*p*-value = 0.0003). Furthermore, the average number of prescriptions of other atypical antipsychotics, such as risperidone, had a substantial decline to half that of their use during the pre-pandemic period (*p*-value = 0.0003). On the other hand, atypical antipsychotic drugs, including clozapine, paliperidone, and quetiapine, indicated no significant variations in their use between the two periods (*p*-value > 0.05). The mean number of prescriptions of the typical antipsychotic haloperidol was approximately 5.00 before the pandemic, and this reduced to 2.64, which is less than half, although this decrease is not significant (*p*-value > 0.05).

The difference in the average consumption of benzodiazepines (clonazepam, diazepam, and lorazepam) and non-benzodiazepines (zolpidem) before and during the pandemic period is not statistically significant (*p*-value > 0.05). For more information, see Table 2.

Table 3 presents the multivariable logistic regression analysis of psychotropic medication use during the COVID-19 pandemic, revealing significantly higher odds for female patients than for male patients (OR = 1.15, 95% CI [1.06–1.26]). Additionally, the patients aged 18–39 were at a significantly higher risk of using psychotropic medications during the pandemic than those aged 40 years and older (OR = 1.65, 95% CI [1.52–1.80]). The use of antidepressants and antipsychotics was significantly lower than that of anxiolytics/sedatives/hypnotics during the pandemic (OR = 0.74, 95% CI [0.65–0.84] and OR = 0.66, 95% CI [0.58–0.75], respectively).

## 4. Discussion

This study included approximately 5000 patients who received various psychotropic drugs between late 2018 and early 2023 in SA. During the COVID-19 pandemic, antidepressant medication use increased relative to that of antipsychotics and anxiolytic/sedative/hypnotic medications. Atypical antipsychotics, benzodiazepines, and SSRIs were the most frequently prescribed medications during the COVID-19 pandemic. Notably, the use of risperidone and olanzapine showed significant differences before and during the pandemic. There was a substantial increase in psychiatric drug use among women and patients <40 years of age. Additionally, the likelihood of using antidepressants and antipsychotics was much lower than that of anxiolytics/sedatives/hypnotics.

A study found that during the pandemic, psychotropic medicines including antidepressants, anxiolytics, and hypnotics were widely prescribed in the US and several European nations, which is consistent with our findings [32,34]. In addition to the antidepressant usage in the European population, it was found that SSRIs were more commonly used than other antidepressants in England and Italy, which is consistent with our findings [29,30]. In addition, the findings of a large international study indicated that people diagnosed with various mental health conditions in the US, South Korea, and Europe were more likely to utilize psychotropic drugs, which is consistent with our findings [35]. All of these studies sourced from the literature suggest the high utilization of different psychotropic drugs during the pandemic compared to that before. This might be related to the influence of the lockdown and the rapid transmission of the virus, which forced individuals to reduce social interactions [20,21,22].

The rise in psychotropic drug prescriptions during the pandemic may have been driven by an increase in mental health issues, including anxiety and depression, in SA and Europe. This is supported by a study that found high rates of anxiety, depression, and stress among 754 individuals in SA, with a significant portion under 45 years old. The prevalence of depression was 43.4%; anxiety, 34.9%; and stress, 36.5% [36]. Another study conducted in SA also reported a substantial increase in depression and anxiety during the pandemic [37]. In Europe, the prevalence of depression ranged from 17% to 30%, and anxiety from 14% to 27% during the pandemic [38,39].

Herein, there were no significant mean differences found in the use of antidepressants, antipsychotics, and anxiolytic/sedative/hypnotic agents, except for olanzapine and risperidone, the use of which declined significantly during the pandemic. In contrast, a study in Brazil found an increase in risperidone prescriptions, which differs from the current findings [40]. This discrepancy may be due to the higher cost of olanzapine in Brazil, leading to a preference for risperidone [41]. The decrease in olanzapine and risperidone use found in the present study could be attributed to safety concerns, prompting healthcare providers to minimize these prescriptions to avoid complications during the pandemic. In addition, a higher prescription rate was noted for quetiapine, a medication in the same class that may be safer and more affordable [40,41,42]. Therefore, this could provide an explanation for the lower prescribing rate of olanzapine and risperidone in our study.

In the present study, women were significantly more likely than men to use psychotropic medications during the pandemic. A similar study corroborated these findings regarding the daily dose of antidepressants, although it focused solely on antidepressants, unlike the present comprehensive study, which included antidepressants, antipsychotics, and anxiolytic/sedative/hypnotic drugs [29]. Studies have shown that women are more likely than men to experience stress, anxiety, or depression during pandemics, which can explain the increased consumption of psychotropic medications among females [20,36]. In the current study, individuals between the ages of 18 and 40 exhibited high usage of psychotropic drugs during the pandemic. This finding aligns with those of a Canadian study, which reported an increased prevalence of antidepressant and antipsychotic drug use among adults under 40 during the pandemic [27]. In SA, individuals aged 18–55 had a higher risk of depression than older persons, whereas the 18–25 age group had a higher risk of anxiety and stress than the other age groups [36]. The increase in mental health disorders among younger people likely contributed to the higher use of psychotropic medications during the study period. The use of antidepressants and antipsychotics compared to that of anxiolytics/sedatives/hypnotics was significantly lower during the pandemic. Although there are no similar studies directly comparing these classes before and during the pandemic, a study found that prescriptions of antidepressants, antipsychotics, and anxiolytics increased significantly during the pandemic [34]. This study examined the use of each investigated drug class to provide guidance to policymakers and healthcare providers. Therefore, the higher use of anxiolytics/sedatives/hypnotics could be due to the increased prevalence of anxiety and sleep problems during the pandemic, likely exacerbated by the detrimental effects of lockdowns on social interaction [43,44]. On the other hand, the high use of anxiolytics/sedatives/hypnotics in our study may have therapeutic consequences, such as dependence, abuse, adverse effects for patients, and an increased risk of falls among the elderly [45,46,47]. Furthermore, the use of anxiolytic/sedative/hypnotic drugs in practice may be associated with a substantial risk of deaths [48]. Therefore, healthcare providers should limit the use of these medications or prescribe them wisely to avoid any complications during or after pandemics.

The strength of the present study lies in its scope as the first large-scale study in SA to examine the use of different psychotropic medications, involving approximately 5000 patients before and during the COVID-19 pandemic. This provides comprehensive insight for healthcare providers and policymakers regarding the use of these medications. However, this study has several limitations. First, specific mental health diagnoses were not included, although it was assumed that each patient had at least one, given that the medications were prescribed by psychiatrists. Second, the data did not include comorbidities, preventing the assessment of the impact of different psychotropic medications on these conditions. Third, we were unable to relate the high usage of anxiolytics/sedatives/hypnotics to the presence of sleep problems since our data did not include the indication for each drug prescription. Fourth, there was a risk of missing bias since certain information on comorbidities and other utilized drugs was not included in our data, which may have had an impact on our study. Fifth, there could be a chance for selection bias since certain psychotropic medications may have another use. Lastly, the study was conducted at a single center in the eastern province of SA, limiting the generalizability of the findings to other regions of the country.

This study has important implications since the COVID-19 pandemic had a catastrophic impact on people’s lives, prompting people to use psychotropic medications. The findings of this study will provide an overview of the most widely utilized psychiatric medications in SA before and during the pandemic. Therefore, the study can also help policymakers establish mental health screening programs in SA to reduce the risk of depression, anxiety, and stress, perhaps lowering the unnecessary use of psychotropic drugs. Finally, future studies should investigate the use of psychotropic medications with mental health conditions. 

## 5. Conclusions

The results of the present study indicate that, during the COVID-19 pandemic, the use of antidepressants was higher than that of other psychotropic medications. Women and individuals aged less than 40 years old were at a higher risk of using psychotropic medications. Finally, the prescription of anxiolytic/sedative/hypnotic medications surpassed that of antidepressants and antipsychotics.

## Figures and Tables

**Table 1:** Baseline characteristics of patients using psychotropic drugs before and during the COVID-19 pandemic.

Characteristic	N * (%) **
Sex	
Male	2177 (44.92)
Female	2669 (55.08)
Age	
18–39	2559 (52.81)
≥40	2287 (47.19)
Antidepressants	
SSRIs	2879 (32.83)
SNRIs	287 (3.27)
Tricyclic	182 (2.08)
Tetracyclic	838 (9.56)
Antipsychotics	
Atypical	3121 (35.59)
Typical	76 (0.87)
Anxiolytics-sedatives/hypotonics	
Benzodiazepines	1267 (14.45)
Nonbenzodiazepines	120 (1.37)

* N = number, ** % = percentage.

**Table 2:** Mean use of psychotropic drugs before and during the COVID-19 pandemic.

Drug	Mean (SD) Before (Q4 2018 to Q1 2020)	Mean (SD) During (Q2 2020 to Q1 2023)	*t*-Value	*p*-Value *
Antidepressants				
Selective serotonin reuptake inhibitors (SSRIs)				
Escitalopram	77.57 (24.51)	20.50 (6.18)	0.65	0.5254
Sertraline	34.64 (13.14)	47.00 (14.55)	−1.82	0.0875
Serotonin and norepinephrine reuptake inhibitors (SNRIs)				
Venlafaxine	12.14 (5.61)	15.73 (6.00)	−1.27	0.2238
Tricyclic				
Amitriptyline	7.71(3.15)	7.18 (2.86)	0.37	0.7156
Clomipramine	3.43 (2.64)	1.64 (1.12)	2.01	0.0613
Tetracyclic				
Mirtazapine	43.50 (14.33)	39.09 (8.35)	0.83	0.4185
Antipsychotics				
Atypical				
Clozapine	3.00 (1.53)	2.73 (1.74)	0.34	0.7387
Olanzapine	41.86 (11.84)	23.55 (5.28)	4.53	0.0003 *
Paliperidone	11.43 (9.45)	8.73(6.10)	0.74	0.4690
Quetiapine	48.36 (16.75)	51.36 (10.46)	−0.47	0.6434
Risperidone	39.00 (15.90)	22.18 (3.25)	3.46	0.0033 *
Typical				
Haloperidol	5.00 (3.92)	2.64 (1.75)	1.77	0.0964
Anxiolytic–sedative/hypotonic				
Benzodiazepines				
Clonazepam	15.36 (11.39)	15.00 (5.69)	0.09	0.9302
Diazepam	2.14 (1.22)	2.73 (1.68)	−0.79	0.4386
Lorazepam	47.43 (30.13)	51.73 (9.58)	−0.45	0.6617
Nonbenzodiazepines				
Zolpidem	4.71 (2.43)	5.00 (2.37)	−0.25	0.8079

* *p*-value < 0.05.

**Table 3:** Multivariable logistic regression analysis of psychotropic drug use during the COVID-19 pandemic.

Predictors	OR *	95% CI **	
Sex (female vs. male)	1.15	1.06	1.26
Age group (18–39 vs. ≥40)	1.65	1.52	1.80
Drug class (antidepressants vs. anxiolytics/sedatives/hypnotics)	0.74	0.65	0.84
Drug class (antipsychotics vs. anxiolytics/sedatives/hypnotics)	0.66	0.58	0.75

* OR = Odds ratio, ** CI: confidence interval; Hosmer and Lemeshow goodness-of-fit test (*p*-value = 0.51).

## Data Availability

The data that support the findings of this study are available from the author upon reasonable request.
